# Multinational landscape of health app policy: toward regulatory consensus on digital health

**DOI:** 10.1038/s41746-022-00604-x

**Published:** 2022-05-11

**Authors:** James A. Diao, Kaushik P. Venkatesh, Marium M. Raza, Joseph C. Kvedar

**Affiliations:** Harvard Medical School, Boston, MA USA

**Keywords:** Health policy, Technology

## Abstract

Due to its enormous capacity for benefit, harm, and cost, health care is among the most tightly regulated industries in the world. But with the rise of smartphones, an explosion of direct-to-consumer mobile health applications has challenged the role of centralized gatekeepers. As interest in health apps continue to climb, national regulatory bodies have turned their attention toward strategies to protect consumers from apps that mine and sell health data, recommend unsafe practices, or simply do not work as advertised. To characterize the current state and outlook of these efforts, Essén and colleagues map the nascent landscape of national health app policies and raise several considerations for cross-border collaboration. Strategies to increase transparency, organize app marketplaces, and monitor existing apps are needed to ensure that the global wave of new digital health tools fulfills its promise to improve health at scale.

In 2020, more than 91,000 digital health apps were released in app stores, averaging 250 new apps per day^1^. At the same time, investors poured $24 billion into the fast-growing digital health market^1^. Although consumer health apps are often associated with wellness and fitness applications, apps targeting specific health conditions—including mental health, diabetes, and cardiovascular disease—now account for half of widely used apps^1^. This expanded role for medical benefit comes with privacy concerns^2^ and the potential for harm^3^. When interest outpaces evidence^4^, principled frameworks and policy are needed for effective stewardship. Centralized programs for review and accreditation would enable clinicians to recommend or prescribe interventions, payors to favor value-based programs, and patients to vet app quality and risks. In their recent article, Essén et al.^5^ systematically document national-level policies for mobile health applications to analyze their regulatory context, evaluation processes, and future directions.

The authors examined national healthcare reports, legislation, published standards, and expert interviews for nine developed countries across Europe, North America, and Asia. Their analysis revealed a patchwork set of programs at varying degrees of maturity. More advanced programs include Germany’s Fast-Track process and Belgium’s mHealthBelgium framework, which provide centralized avenues for market access and reimbursement approval. Emerging programs include the Digital Technology Assessment Criteria (DTAC) in England and the Software Precertification Pilot Program (Pre-Cert) in the US, which will inform forthcoming regulatory models. Less centralized programs include the Swedish Accreditation Agency, which certifies third-party organizations to evaluate apps based on common criteria. In most other countries, regulatory policy is divided among regional recommendations in need of consolidation, consistent with trends from earlier studies^6^. Among interviewed individuals involved in developing frameworks, most anticipate sustained progress toward centralized directories of approved apps and platform-based curation of market access.

Digital health is an amorphous and rapidly evolving space that presents several challenges for traditional regulatory frameworks. These include the vast number of available apps, their instantaneous global reach, and rapid changes permitted by software updates^7^. Despite these unique attributes, lessons from other regulatory strategies may be useful. For example, “nutrition facts”-inspired labels for health apps, such as the model by the International Organization for Standardization (ISO)^8^, may increase transparency on privacy, security, and efficacy^9^. Centralized curation of a consumer-friendly app marketplace, similar to the Health Insurance Marketplace created by the Affordable Care Act, may empower consumers to compare and assess competing options^10^. Risk-based post-market surveillance, increasingly pursued in drug safety monitoring, may also offer a scalable strategy. An analysis of health apps in the Google Play Store showed that the most popular 1% of apps account for >80% of downloads while the least popular 80% garnered <1% of downloads^1^. This skew suggests that post-market evaluation remains feasible despite the daunting multitude of health apps, if agencies can target the most popular and highest risk services.

Commercial entities may also help organize the sprawl of new digital health products. Comprehensive apps like Apple Health aggregate information across health records and third-party apps into a single secure and interoperable location. Companies like the Organisation for the Review of Care and Health Apps (ORCHA) assist governments with accreditation, help app developers meet compliance standards, and curate digital health services for patients and providers. Some, like Xealth, go one step further to bundle compatible digital services into a single platform marketed to providers. While less scalable than direct-to-consumer models, commercial intermediaries provide innovative alternatives that may integrate more readily with existing care pathways and systems.

Implementation and standardization of any strategy will require coordination between multiple stakeholders, including regulatory agencies, app developers, payors, and providers. But with the right incentives and guardrails, a vision emerges for the future of mobile health apps. Clinicians and patients could view lists of approved apps that are supported by data, certified for privacy protections, covered by their insurance, and interoperable with their electronic health record. Rapid software updates could implement new guidelines, patch software errors, and respond to changes in behavior. Cross-border quality control agreements could reinforce trust as these apps are deployed around the globe. With an eye toward this future, Essén et al. offers a comprehensive review of the current state of health app policy and its gradual progress toward international consensus. As values are codified in frameworks and frameworks translated into legislation, we believe the continuing surge in health apps can be effectively guided toward its potential to deliver patient-centered, technology-enabled, and value-based care.
